# Temperature affects maximum H-reflex amplitude but not homosynaptic postactivation depression

**DOI:** 10.1002/phy2.19

**Published:** 2013-06-28

**Authors:** Sébastien Racinais, Andrew G Cresswell

**Affiliations:** 1 Aspetar, Qatar Orthopaedic and Sports Medicine HospitalDoha, Qatar; 2 The University of Queensland, Centre for Sensorimotor Neuroscience, School of Human Movement StudiesBrisbane, Australia

**Keywords:** Exercise, hyperthermia, muscle, neuromuscular function, spinal reflex

## Abstract

This study aimed to determinate the effect of hyperthermia on transmission efficacy of the Ia-afferent spinal pathway. Recruitment curves of the Hoffman reflex (H-reflex) and compound motor potential (M-wave) along with homosynaptic postactivation depression (HPAD) recovery curves were obtained in 14 volunteers in two controlled ambient temperatures that resulted in significantly different core temperatures (CON, core temperature ∼37.3°C; and HOT, core temperature ∼39.0°C). Electromyographic responses were obtained from the soleus (SOL) and medial gastrocnemius (MG) muscles following electrical stimulation of the tibial nerve at varying intensities and paired pulse frequencies (0.07–10 Hz). Results showed that maximal amplitude of the H-reflex was reached for a similar intensity of stimulation in CON and HOT (both muscles *P* > 0.47), with a similar associated M-wave (both muscles *P* > 0.69) but was significantly decreased in HOT as compared to CON (all *P* < 0.05), whether expressed in absolute terms (−50% in SOL, −32% in MG) or when normalized to the maximum M-wave (−23% in SOL, −32% in MG). The HPAD recovery curve was not affected by the elevated core temperature (both muscles *P* > 0.23). Taken together, these results suggest that hyperthermia can alter neuromuscular transmission across the neuromuscular junction and/or muscle membrane as well as transmission efficacy of the Ia-afferent pathway, albeit the latter not via an increase in HPAD.

## Introduction

Temperatures above 50°C have been observed in various populated areas within Africa, Asia, and the Middle East. Passive hyperthermia in high temperature conditions is known to reduce neural drive to human skeletal muscle (Morrison et al. 2004; Thomas et al. 2006; Racinais et al. 2008). This reduction is partly due to altered neuromuscular transmission as evidenced by a decrease in an electrically evoked M-wave, Hoffman reflex (H-reflex), and V-wave (Racinais et al. 2008).

The amplitude, duration, area, and latency of a compound action potential have all been reported to be negatively correlated to skin temperature (Bolton et al. 1981) with the reduction in duration and amplitude of a response being attributed to a shortening of the time that the voltage-gated sodium channels remain open when temperature increases (Rutkove et al. 1997). However, some in vitro studies suggest that the decrement in the amplitude of electrically evoked potential may originate from a failure of synaptic transmission at high temperatures (Kelty et al. 2002). Indeed, it has previously been observed that at 22°C all synapses produced one or more quantal events for each nerve impulse without failure, but as temperature increased the quantal event was reduced until transmission failed when the nerve temperature reached 35°C (Karunanithi et al. 1999). However, these in vitro observations have to be interpreted with caution as M-wave amplitudes have been shown to decrease, but not disappear in vivo, even with human muscle temperatures exceeding 40°C (Racinais and Girard 2012). Additionally, it has been suggested that function of the human neuromuscular junction is minimally influenced by temperature, due to a high safety factor with more acetylcholine released than necessary for a given stimulus (Rutkove 2001).

As such, it is still undetermined if the reduced amplitude in electrically evoked potentials in vivo is due to transmission failure or some other neural factor. Whereas most of the previous studies in humans have recorded single evoked potentials (Bolton et al. 1981; Rutkove et al. 1997; Dewhurst et al. 2005; Racinais et al. 2008; Gaoua et al. 2011; Racinais and Girard 2012), synaptic limitation might become more evident in the presence of repeated stimuli. Indeed, even in a temperate environment, homosynaptic postactivation depression (HPAD) induced by multiple stimuli limits the ability of a spinal motoneuron to be repeatedly activated via Ia-afferent input (Hultborn et al. 1996; Pinniger et al. 2001; Nordlund et al. 2004).

Therefore, the aim of this study was to investigate the effect of hyperthermia on the ability of the motoneurone to repeatedly discharge. If HPAD is accentuated by hyperthermia, it may limit motoneurone firing rate and therefore explain why neural drive is not able to compensate for the shortening of a muscle twitch which is known to occur at high temperatures (Todd et al. 2005). Moreover, observing a decrease in synaptic transmission efficacy in humans would suggest the existence of possible countermeasure as animal models have suggested that thermal preconditioning or exogenous Heat Shock Protein preserves synaptic transmission during thermal stress (Kelty et al. 2002).

## Methods

### Participants

Fourteen healthy participants (seven males and seven females, age 29–34 year, weight 67.8 ± 10.6 kg and height 170 ± 6 cm) completed the study. None of the participants suffered from injuries at the time of the experiment and they were required to avoid all vigorous activity for 24 h preceding the testing.

The project was approved by the Aspetar scientific committee (approval CMO/085,011/GS/ct) and by the Shafallah ethics committee (external institutional review board (IRB), approval 2011-005). The procedures complied with the Declaration of Helsinki regarding human experimentation. Written informed consent was obtained from all the participants before the beginning of the testing.

### Procedures

Following a familiarization trial, participants underwent experimental trials using the same protocol in two temperature conditions, control (CON, room set at 24°C and 35% relative humidity [RH]) and hot (HOT, room set at 50°C and 35% RH) within an environmental room (Tescor, Warminster, PA). Trials were performed in a counter-balanced order, at the same time-of-day on different days and with subjects wearing the same minimal clothing (i.e., shorts and T-shirt).

Prior to each trial, participants rested for 1 h (laboratory at ∼24°C) and drank 500 mL of water while being equipped with rectal and skin temperature probes, a heart rate (HR) monitor, and electromyographic (EMG) surface electrodes (see below for details). Participants were asked to provide a urine sample. If the specific gravity of the sample (URC-NE, Atago, Tokyo, Japan) was above 1.020 g/mL the trial was postponed and the participants were required to drink more water.

Participants then entered the environmental room and walked on a motorized treadmill for 10 min at 4 km/h, which was immediately followed by 45–60 min of rest in a seated position, corresponding to the time needed to reach a core temperature of 39°C in the HOT trial (Racinais et al. 2008). This was done prior to performing the neurophysiological assessments, which were performed on the soleus (SOL) and medial gastrocnemius (MG) muscles of the right leg. For these measurements the participants remained in the environmental room and were in a seated position with their ankle and the knee at 90° and 100°, respectively, and their foot securely strapped to a custom-made foot plate. During the HOT trial, core temperature was maintained at ∼39°C by adjusting the room temperature as necessary in the range of 46–50°C with a RH of 35%.

### Measurements

#### Physiological monitoring

Rectal temperature, skin temperature, and HR were continuously monitored using a rectal probe (Ellab, Hilleroed, Denmark; inserted 15 cm beyond the anal sphincter), a surface thermistor (Ellab, Hilleroed, Denmark; over SOL and MG), and a chest strap (Polar, Electro OY, Kempele, Finland), respectively. Body mass and urine specific gravity (USG) were recorded before and after the testing session.

#### Electromyography

Bipolar EMG signals were recorded over the muscle belly of SOL using Ag/AgCl electrodes (Ambu Blue sensor T, Ambu A/S, Denmark) with a recording diameter of 9 mm and an interelectrode distance of 3 cm. A pseudo-monopolar EMG signal was recorded from MG, with one electrode located on the muscle belly and one electrode on the distal portion of the Achilles tendon. All EMG signals were recorded using MP35 hardware (Biopac Systems Inc., Santa Barbara, CA) and dedicated software (BSL Pro Version 3.6.7, Biopac Systems Inc., Santa Barbara, CA). The EMG signal was amplified (gain = 1000 for bipolar and 200 for pseudo-monopolar recordings), filtered (30–1000 Hz), and recorded at a sampling frequency of 5 kHz. Before electrode placement, the skin was shaved and washed to remove surface layers of dead skin, hair, and oil; and a reference electrode was placed over the patella.

### Protocol

#### Evoked potentials

Percutaneous stimulations (400 V, rectangular current pulse of 1 msec) were delivered by a constant current stimulator (Digitimer DS7AH, Digitimer, Hertfordshire, England) to the tibial nerve. The cathode (diameter = 9 mm, Ambu Blue sensor T, Ambu A/S, Denmark) was located in the popliteal fossa (with constant pressure supplied by a strap) and the anode (5 × 9 cm) located slightly distal to the patella. Placement of the electrodes were marked with a “permanent” marker and kept constant throughout the experimental days. Initially, the current was progressively increased in small increments until there was no further increase in the peak-to-peak amplitude of the electrophysiological M-wave (M_MAX_). Thereafter, the current was adjusted in smaller increments to determine the maximal peak-to-peak amplitude of the H-reflex (H_MAX_). Current was then adjusted to an intensity that resulted in a H-reflex of ∼50% of its maximal amplitude for subsequent HPAD measures.

### Data analysis

A HPAD “recovery curve” was produced by evoking paired pulse H-reflexes (current intensity set to elicit ∼50% of H_MAX_). Five or six paired pulses were evoked at each of the following frequencies: 10, 8, 6, 4, 3, 2, 1.5, 1, 0.8, 0.6, 0.4, 0.2, and 0.1 Hz, in a counter-balanced order (with a minimum of 20 sec of rest). The peak-to-peak amplitudes of the two H-reflexes were measured and the second reflex response (H_2_) was expressed as a percentage of the first reflex response (H_1_) within the same pair. For each test frequency, the lowest and highest values were deleted and the remaining three or four values averaged. A “recovery curve” was draw to represent the amplitude of H_2_ as a percentage of H_1_ in function of the time separating the two stimulations.

### Statistics

H_MAX_ and M_MAX_ data were compared in CON versus HOT with a one-way repeated measures analysis of variance (ANOVA). HPAD recovery was analyzed using a two-way repeated-measure ANOVA to study the effects of pulse frequency, condition (CON vs. HOT), and their potential interaction.

The level of statistical significance was set at *P* ≤ 0.05. All data are presented as means ± standard deviation (SD) in the text and standard error of the mean (SEM) in figures.

## Results

### Physiological responses

Core (39.0 ± 0.2°C vs. 37.3 ± 0.4°C) as well as skin (39.8 ± 0.8°C vs. 29.5 ± 0.4°C) temperatures were both significantly higher during the testing in HOT than CON, respectively (both *P* < 0.001). HR was also higher in HOT than CON (102 ± 13 vs. 60 ± 11 bpm, *P* < 0.001).

There was no evidence of dehydration during the HOT and CON trials (USG: 1.012 ± 0.008 vs. 1.012 ± 0.005 g/mL, *P* = 0.98) and body mass did not change significantly during any trial (−0.2 ± 0.9 kg in HOT vs. −0.1 ± 0.3 kg in CON; *P* = 0.64).

### H-reflex and M-wave amplitude

**Figure 1:**
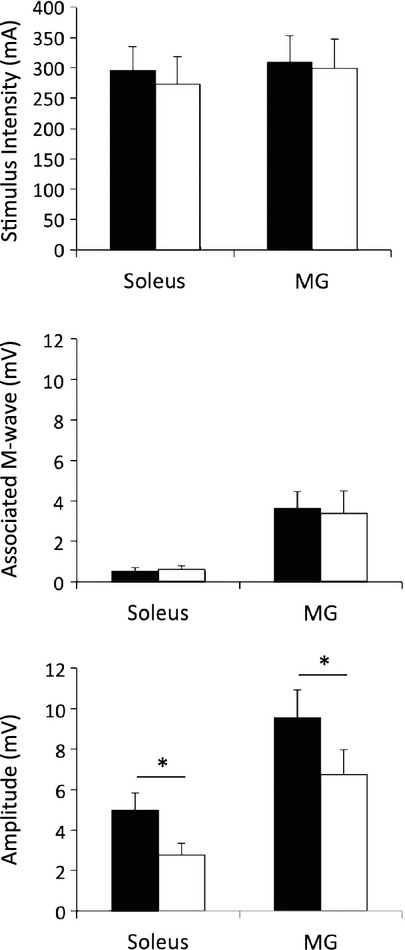
Maximal Hoffman reflex (H-reflex). Data obtained from Soleus (SOL) and medial gastrocnemius (MG) muscles in CON (filled columns) and HOT (open columns) conditions. Data represents the intensity of the electrical stimulation inducing a maximal H-reflex (top graph), the mean amplitude of the associated M-wave (middle graph) and the maximum H-reflex amplitude (bottom graph). *P < 0.05.

H_MAX_ was reached for a similar intensity of stimulation in CON and HOT (both muscles *P* > 0.47; Fig. 1), with a similar associated M-wave (both muscles *P* > 0.69; Fig. 1) but was significantly lower in HOT as compared to CON (SOL −50 ± 13%, MG −32 ± 20%, both muscles *P* < 0.005; Fig. 1).

M_MAX_ was lower in HOT than CON for SOL (7.28 ± 3.02 vs. 10.15 ± 5.45 mV, *P* = 0.035) but not MG (26.17 ± 5.43 vs. 26.44 ± 5.68 mV, *P* = 0.91). The changes in M_MAX_ (SOL −15%, GM 0.6%) did not account for the observed reduction in H_MAX_ (SOL −50%, MG −32%; Fig. 1) as H_MAX_:M_MAX_ was significantly lower in HOT than CON for both SOL (−23%, 41.1 ± 24.6 vs. 57.4 ± 30.0%, *P* = 0.046) and MG (−32%, 26.3 ± 14.5 vs. 37.6 ± 15.8%, *P* = 0.001).

The latency of both M-wave and H-reflex for both muscle were all significantly shorter in HOT than CON (M-wave 14 ± 2 vs. 16 ± 2 msec, H-reflex 38 ± 2 vs. 41 ± 3 msec, all *P* < 0.001).

### Homosynaptic postactivation depression

**Figure 2:**
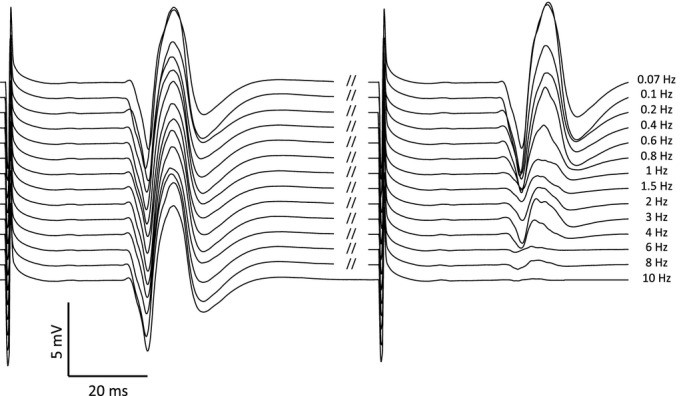
Paired Hoffman reflexes (H-reflexes) recorded at different stimulations frequencies. Data from one pair of H-reflex obtain from medial gastrocnemius (MG) at each stimulation frequency in a representative participant in the control condition.

**Figure 3:**
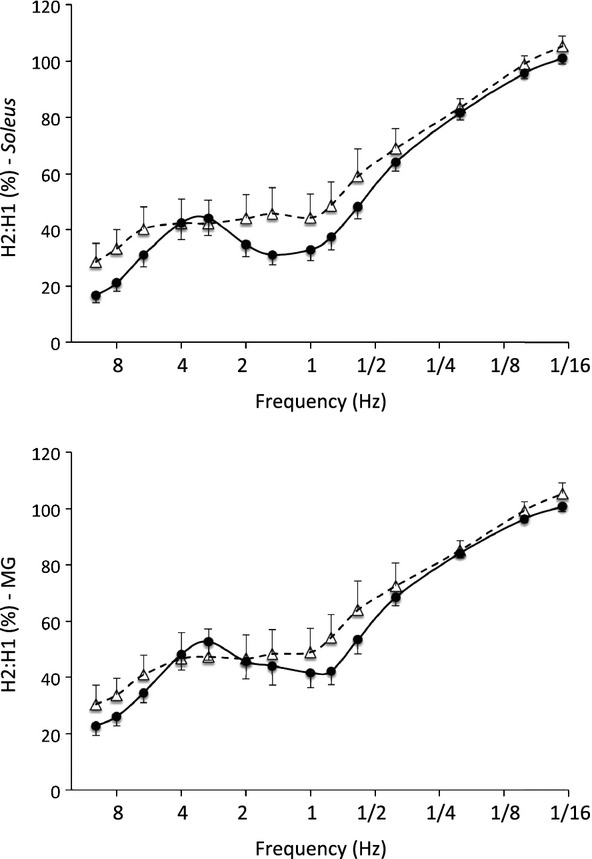
HPAD recovery curve. Data represent the second Hoffman reflex (H-reflex) (H_2_) expressed as a percentage of the first (H_1_) during the paired pulse stimulation at different frequencies (logarithmic scale). Data obtained from soleus (top graph) and medial gastrocnemius (bottom graph) muscles in CON (solid line, filled circle) and HOT (dashed line, open triangle) conditions. There was a significant effect of time but no effect of condition or interaction.

An example of paired H-reflexes at different frequencies of stimulation is displayed in Figure 2. The amplitude of the first H-reflex during the doublet was not affected by the stimulation frequency (*P* > 0.24 for both SOL and MG). The expression of the second H-reflex as a percentage of the first for different interstimulus intervals (HPAD; Fig. 3) showed a consistent recovery with decreased stimulation frequency (i.e., increasing the interstimulus interval) (*P* < 0.001 for both SOL and MG). HPAD recovery was, however, not affected by the ambient conditions (*P* > 0.28 for both muscles between CON and HOT; Fig. 3).

## Discussion

The main finding of the study was that passive hyperthermia decreased the amplitude of the maximum H-reflex (both absolute and normalized to M_MAX_). However, postactivation depression in H-reflex amplitude induced by repeated stimuli was not affected by increased temperature conditions.

### H-reflex amplitude is affected by core temperature

Local warming of the leg (soleus skin temperature ∼36.6°C) has previously been reported to alter maximum H-reflex and M-wave amplitudes, but not H_MAX_ when normalized to its respective M_MAX_ (Dewhurst et al. 2005). Those results suggest that an increase in local skin and muscle temperature does not affect the efficacy of the Ia-afferent/motoneurone pathway. Conversely, an increase in core temperature (i.e., passive hyperthermia) has been shown to reduce both H-reflex and V-wave (an electrophysiological variant of the H-reflex) amplitudes, even when normalized to their corresponding M-waves (Racinais et al. 2008). Given that such decrease persisted when the head was maintained artificially cooler (Racinais et al. 2008) we hypothesized that an increase in core temperature would affect the efficacy of the Ia-afferent/motoneurone pathway. According our hypothesis, data showed that the maximum H-reflex amplitude was lower when core temperature was ∼39.0°C (i.e., passive hyperthermia), as compared to temperate conditions with a core temperature of ∼37.3°C. In the current experiment, the maximal M-wave was also slightly lower in HOT than CON for SOL but not MG. When H_MAX_ was expressed relative to M_MAX_, like the nonnormalized result the response for both SOL and MG was reduced in HOT compared to CON conditions. These observations confirm that an increase in core temperature can alter the responsiveness of the Ia-afferent motoneurone pathway.

In addition, the current data showed for the first time that, despite differences in skin and core temperatures, H_MAX_ was achieved with a similar intensity of stimulation in both CON and HOT and with similar associated M-waves. This further suggests that the decrease in H_MAX_ is linked to changes at the spinal level. Interestingly, the decrement in normalized reflex waves (i.e., H-reflex/M-wave) is not of bigger amplitude after a maximal exercise in hot than after a maximal exercise in temperate environment (Racinais and Girard 2012) suggesting that the changes due to temperature do not cumulate to the previously observed changes due to exercise-induced fatigue (Duchateau and Hainaut 1993; Racinais et al. 2007). Such change may include alterations at either or both of the presynaptic and the postsynaptic terminal (Racinais and Oksa 2010). For example, the reduction in reflex size could result from an increase in presynaptic inhibition mediated by sources such as group III and IV afferents (Bigland-Ritchie et al. 1986; Woods et al. 1987; Garland and McComas 1990; Garland 1991; Duchateau et al. 2002; Avela et al. 2006) as animal model have shown an increase in the discharge rate of group III and IV muscle afferents in response to an increase in muscle temperature (Hertel et al. 1976; Kumazawa and Mizumura 1977). The decrement in H-reflex observed in the current study might also be related to a failure of the synapse to depolarize the postsynaptic element due to a more generalized reduction in transmitter release from the presynaptic terminal for each Ia volley or a failure of the synaptic transmission at high temperatures (Karunanithi et al. 1999; Kelty et al. 2002).

### The HPAD is not affected by passive hypethermia

The ability of the spinal synapse to repeatedly discharge and activate the postsynaptic element was tested using paired pulse H-reflexes (i.e., HPAD calculated as H_2_:H_1_) to investigate whether changes in core temperature affected Ia-afferent synaptic transmission. Previous studies have shown that that the H-reflex interstimulus interval has a significant effect on the amplitude of the second H-reflex relative to the first (Hultborn et al. 1996). The second H-reflex is almost extinguished with interpulse intervals of a few milliseconds but systematically recovers as the pulse interval increases and in most cases full recovers at interpulse intervals approaching 10 sec. The mechanism behind the long-term depression is likely an activation history dependent, reduced probability of transmitter release from the Ia fibers. Like earlier studies (Crone and Nielsen 1989), our data showed a significant reduction of H_2_ at high stimulation frequencies followed by a progressive recovery as the time between stimuli lengthened. This mechanism might partly account for the immediate postexercise H-reflex depression (e.g., Duchateau and Hainaut 1993), but not for the decrements persisting 30 min after prolonged exercise (Racinais et al. 2007). Given that hyperthermia is an ongoing factor, we hypothesized it may continuously affect synaptic properties and as such, HPAD recovery may take longer in the HOT condition. Interestingly, our data showed that neither the time nor amplitude properties of HPAD recovery were affected by the ambient conditions. Of course synaptic properties were already altered in the HOT conditions as the first stimulus of the pair (H_1_) in that condition showed a significant decrease in H_MAX_ compared to CON. However, given that HPAD recovery is calculated by expressing H_2_ as a percentage of H_1_, any initial alterations that were affecting H_1_ are likely to also be applicable to H_2_. Taken together, these results showed that passive hyperthermia alters synaptic transmission at the spinal level but does not further alter the relative kinetics of recovery with repeated discharges.

### Limitations and perspectives

Decrements in electrically evoked action potential amplitudes have been reported both in vitro and in vivo (see Rutkove 2001; Racinais and Oksa 2010 for reviews). These decrements could be explained by either a shortening of the time that the voltage-gated sodium channels remain open with increasing temperature, leading to a decrease in the amplitude, duration, and area of a single axon potential (Bolton et al. 1981; Rutkove et al. 1997) or by a failure of synaptic transmission at high temperature (Karunanithi et al. 1999; Kelty et al. 2002). Surprisingly, in the current experiment, passive hyperthermia reduced the M-wave amplitude in the SOL but not MG. This decrement in M-wave might also have been affected by changes occurring between the activated muscle and the recording surface electrodes such as cutaneous vasodilatation or changes in skin-electrode properties (Bell 1993), and the different responses observed in SOL and MG might be related to the different technique used (e.g., bipolar vs. monopolar EMG). It is worthwhile to note that a similar observation has recently been reported in another study with a decrement of M-wave amplitude in the SOL in hot environment but not in the Vastus Lateralis and Rectus Femoris muscles (Racinais and Girard 2012). The current experiment was not designed to determine the cause for this variability, however, future studies could investigate the possibility that response to hyperthermic conditions may be muscle specific and used intramuscular EMG to account for any changes occurring at the level of the skin-electrode pair.

## Conclusion

The current experiment used electrically evoked H-reflexes to investigate the responsiveness of the Ia-afferent pathway in different temperature conditions. Our data showed that the maximal H-reflex was obtained for a similar intensity of stimulation but was of lower amplitude when core temperature was elevated. This suggests that passive hyperthermia alters the responsiveness of the pathway (synaptic transmission). While such conditions altered transmission of a single action potential, it does not appear to alter long-term synaptic depression from repeated discharges.
